# Modulation of pain perception by transcranial magnetic stimulation of left prefrontal cortex

**DOI:** 10.1007/s10194-011-0322-8

**Published:** 2011-02-25

**Authors:** Filippo Brighina, Marina De Tommaso, Francesca Giglia, Simona Scalia, Giuseppe Cosentino, Angela Puma, Maristella Panetta, Giuseppe Giglia, Brigida Fierro

**Affiliations:** 1 Dip di Biomedicine Sperimentali e Neuroscienze Cliniche (BioNeC), University of Palermo, Via G. La Loggia, 1, 90129 Palermo, Italy; 2 Dip di Scienze Neurologiche e Psichiatriche, University of Bari, Bari, Italy

**Keywords:** Capsaicin, Pain, Prefrontal cortex, Magnetic stimulation

## Abstract

Evidence by functional imaging studies suggests the role of left dorsolateral prefrontal cortex (DLPFC) in the inhibitory control of nociceptive transmission system. Repetitive transcranial magnetic stimulation (rTMS) is able to modulate pain response to capsaicin. In the present study, we evaluated the effect of DLPFC activation (through rTMS) on nociceptive control in a model of capsaicin-induced pain. The study was performed on healthy subjects that underwent capsaicin application on right or left hand. Subjects judged the pain induced by capsaicin through a 0–100 VAS scale before and after 5 Hz rTMS over left and right DLPFC at 10 or 20 min after capsaicin application in two separate groups (8 subjects each). Left DLPFC-rTMS delivered either at 10 and 20 min after capsaicin application significantly decreased spontaneous pain in both hands. Right DLPFC rTMS showed no significant effect on pain measures. According to these results, stimulation of left DLPFC seems able to exert a bilateral control on pain system, supporting the critical antinociceptive role of such area. This could open new perspectives to non-invasive brain stimulation protocols of alternative target area for pain treatment.

## Introduction

Chronic pain represents a relevant medical condition with detrimental effects on life quality and socio-economical state. Patients with chronic pain may not respond positively to standard pharmacological therapies and may require other alternative approaches to relieve symptoms. In 1991, Tsubokawa et al. [1] reported efficacy of motor cortex stimulation (MCS) by dural implanted electrodes for treatment of chronic, central, drug-resistant neuropathic pain on 12 patients. Since then, a consistent bulk of evidence showed this approach as being effective for pain control in several patients [2–4]. On the basis of MCS results, the introduction of transcranial magnetic stimulation (TMS) has increased the opportunities to easily and painlessly perform effective human cortex stimulation. Moreover, the use of repetitive stimulation (rTMS) is also able to induce long-lasting plastic changes, whose effects depend on the stimulation frequency used: increased or decreased excitability following low or high-frequency TMS, respectively [5]. Motor cortex rTMS for control of pain was first applied by Migita et al. [6] that showed pain reduction in two patients treated by low-frequency (<0.2 Hz) rTMS. Since then, evidence of potential effect of motor cortex rTMS on pain control has been reported on patients [7–10] as well as on pain model in healthy subjects [11–13]. The great majority of TMS studies [7–10] focused on motor cortex and this site has been considered the optimal area for control of neuropathic pain also by the EFNS Guidelines on Neurostimulation Therapy [14]. The reasons why stimulation of motor cortex is effective in the treatment of pain are not yet completely known. In the study by Tamura et al. [12], a SPECT analysis under the condition of 1 Hz rTMS of right motor cortex (M1), demonstrated a significant relative rCBF decrease in the right medial prefrontal cortex (MPFC), and a significant increase in the caudal part of the right anterior cingulate cortex (ACC) both correlating with pain reduction [12]. This could mean that motor cortex stimulation could indirectly act on pain through the deactivation of MPFC and activation of ACC.

Neurosurgical observations and functional imaging studies have identified a matrix of structures in the brain “pain matrix” that responds to noxious stimuli in which authors identified a clear division of functions between sensory-discriminative and affective responses [15]. Interesting results on pain syndromes also came by stimulation of dorsolateral prefrontal cortex (DLPFC). First indicated as a valid stimulation area for the treatment of depressive states [16, 17], recently the DLPFC has also been considered a potential target for nociceptive control [18].

Functional imaging studies [19, 20] showed that DLPFC activation is temporally related to amelioration of pain sensation in a model of acute pain induced by capsaicin.

Since then DLPFC rTMS has been found effective for the treatment of pain conditions such as chronic migraine [21] and fibromyalgia [22].

In agreement with these results, recent studies showed that DLPFC stimulation can be effective in pain control significantly increasing the threshold for thermal and pain sensation in healthy subjects [23–25] and reducing clinical symptoms and the need for analgesic drugs on postoperative and neuropathic pain [26, 27]. Moreover, the role of DLPFC on pain control has been recently investigated with another non-invasive brain stimulation technique: transcranial direct current stimulation (tDCS) in healthy subjects [28] and in patients with fibromyalgia [29].

On such grounds, the aim of our study was to explore the analgesic effect of DLPFC rTMS in healthy subjects, using a model of acute pain induced by topical application of capsaicin that is known to activate nociceptive primary afferent C-fibers with minimal contributions from other somatosensory modalities [30, 31].

## Materials and methods

We explored the effects of left and right DLPFC rTMS over pain induced by capsaicin in a group of healthy subjects. Sixteen healthy, right handed, drug-free volunteers participated in the study. All subjects were unaware of the study aim and had never experienced magnetic stimulation before. They all signed an informed consent and the study was conducted according to the Declaration of Helsinki.

### Application of capsaicin and pain measures

**Fig. 1:**
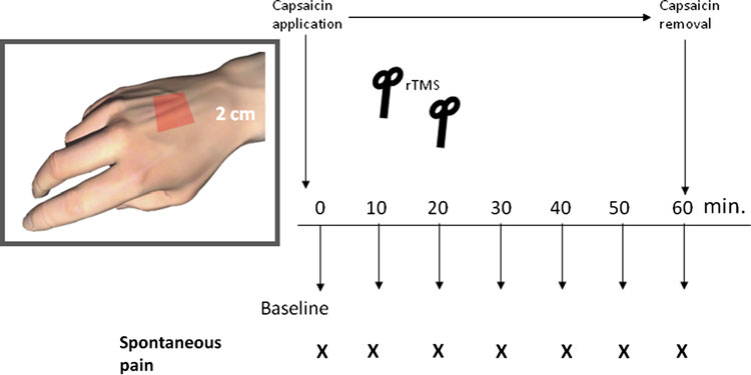
Site of capsaicin application and flow chart of the experiment: times for pain measurements and rTMS delivering (10 and 20 min after capsaicin application)

Capsaicin (Dolpyc Teopharma 3%) was applied over the dorsal surface of the right or left hands on a square area of 2 × 2 cm (see Fig. 1). Subjects judged the pain induced by capsaicin through a 0–100 point visuoanalogic scale (VAS) during application of capsaicin every 10 min for 60 min (till capsaicin removal).

### Transcranial magnetic stimulation

rTMS was delivered through a water-cooled figure of eight coil, powered by a Cadwell High Speed Magnetic Stimulator. According to Pascual Leone et al. [17], DLPFC was localized on the scalp 5-cm anterior to the hot spot for the contralateral abductor pollicis brevis (APB) muscle. To check the correspondence of the stimulated point on the scalp with the targeted brain area (DLPFC), we performed a 3D graphical elaboration of MRI scans to localize DLPFC in seven subjects. Then, a virtual marker was positioned and reached in the real subject’s head using the Brainvoyager QX neuro-navigation system. In all subjects, the corresponding site on the scalp was found to be very close or overlapping the point where rTMS was performed (5 cm anterior to the hotspot for APB muscle).

Motor threshold (MT) was measured at the hotspot of the right APB muscle as the minimum stimulus intensity able to elicit a motor evoked potential (MEP) of at least 50 μV in 5 or more of 10 consecutive stimulations. High-frequency (hf) rTMS at 5 Hz rate was delivered in sessions consisting of 1,800 stimuli each, divided in 12 trains (150 stimuli, 30 s duration each), given at 90% MT intensity and separated by 10-s pause. rTMS was delivered over left and right DLPFC.

### Experimental paradigm

All subjects (8 M/8F; mean age 32.9 ±7, range 28–48 years) underwent hf rTMS over left and right DLPFC after capsaicin application on right and left hand. In eight of them (Experiment 1A), rTMS was delivered at 10 min and in the remaining eight (Experiment 1B) at 20 min after capsaicin application. In each group, subjects underwent six different experimental sessions (3 conditions: capsaicin alone, capsaicin + left and capsaicin + right DLPFC rTMS × 2 hands) with at least 48-h interval between the sessions. In each session, pain measures were evaluated every 10 min for 60 min after capsaicin application. The order of the sessions was randomized across subjects.

### Statistical analysis

Repeated measures analysis of variance (ANOVA) has been used to compare measures of pain across all experimental sessions with and without rTMS. For Experiment 1, the following factors were taken into consideration: (1) between subjects: time of application of rTMS (2 levels: 10 and 20 min); (2) within subjects: hand (2 levels: right and left), condition (3 levels: capsaicin alone, capsaicin + left and capsaicin + right DLPFC rTMS), time of detection of pain measures (7 levels = baseline, 10′, 20′, 30′, 40′, 50′, 60′). Newman Keuls test was performed for post hoc comparisons (see “Results” for details).

## Results

rTMS was well tolerated, capsaicin application only caused local rush and a light to moderate burning sensation.

**Fig. 2:**
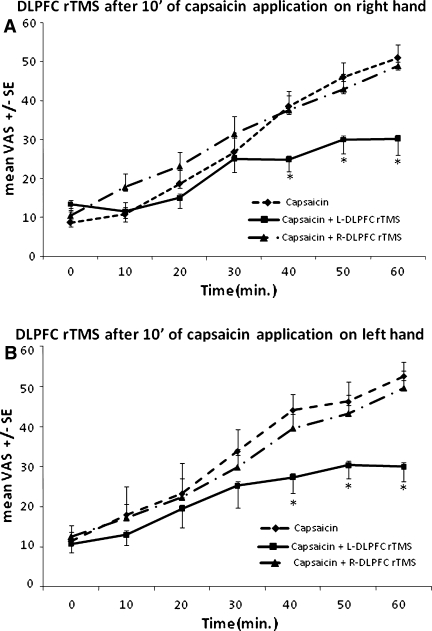
Effects of left and right DLPFC rTMS (delivered at 10 min after capsaicin application) on pain: changes in VAS values (mean ± SE) across different times [baseline (0) to 60 min] in conditions without and with rTMS with capsaicin over the right (**a**) and left hand (**b**); *asterisk* indicates significant differences (*p* < 0.05) in L-DLPFC rTMS with respect to analog time points of the other conditions

**Fig. 3:**
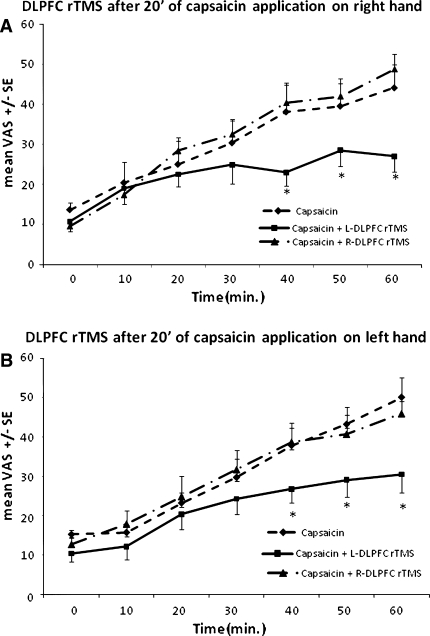
Effects of left and right DLPFC rTMS (delivered at 20 min after capsaicin application) on pain: changes in VAS values (mean ± SE) across different times [baseline (0) to 60 min] in conditions without and with rTMS with capsaicin over the right (**a**) and left hand (**b**); *asterisk* indicates significant differences (*p* < 0.05) in L-DLPFC rTMS with respect to analog time points of the other conditions

ANOVA for repeated measures with: conditions (3 levels: capsaicin alone, capsaicin + left and right DLPFC rTMS), times (7 levels 0–60 min.), hand (2 levels right and left) as within-subject factors and rTMS timing (2 levels 10, 20 min) as between-subject factors showed a significant main effect for the factors times *F*(6,84) = 174,20; *p* < 0.0001; conditions *F*(2,28) = 9,11; *p* < 0.0009; and for the interaction conditions × times *F*(12,168) = 8,30; *p* < 0.00001. No significant main effects were observed neither for hands nor for rTMS timing. Newman–Keuls post hoc test showed that (1) in condition without rTMS (capsaicin alone), spontaneous pain significantly increases on both hands after 20 min of capsaicin application and further increased up to 60 min (see Table 1 for *p* values); (2) the left DLPFC rTMS induces a significant pain reduction with respect to the capsaicin alone condition at 40, 50 and 60 min (*p* values are reported in Table 2) after capsaicin application on both hands and regardless of rTMS time delivering (10 or 20 min). No significant changes in pain measures were observed after right DLPFC rTMS on both hands (see Figs. 2a, b, 3a, b).

**Table 1:** *p* values of post hoc comparisons of VAS values at different times (20–60 min) after capsaicin application with respect to baseline (time 0) in condition without rTMS in the two groups

Minutes	Right hand		Left hand	
	10′ rTMS	20′ rTMS	10′ rTMS	20′ rTMS
20	<0.05	<0.01	<0.05	<0.05
30	<0.001	<0.0001	<0.0001	<0.01
40	<0.0001	<0.0001	<0.0001	<0.0001
50	<0.001	<0.0001	<0.0001	<0.0001
60	<0.0001	<0.0001	<0.0001	<0.0001

**Table 2:** *p* values of post hoc comparisons of VAS changes after 10′ and 20′ rTMS over left DLPFC at 40, 50 and 60 min after capsaicin application, with respect to corresponding VAS values in capsaicin alone condition and after 10′ and 20′ rTMS over right DLPFC

	Right hand		Left hand	
	Capsaicin alone versus L-DLPFC rTMS	R- versus L-DLPFC rTMS	Capsaicin alone versus L-DLPFC rTMS	R- versus L-DLPFC rTMS
10′ rTMS (min)				
40	<0.05	<0.05	<0.01	<0.05
50	<0.01	<0.05	<0.01	<0.05
60	<0.0001	<0.0001	<0.0001	<0.0001
20′ rTMS (min)				
40	<0.05	<0.001	<0.05	<0.05
50	<0.05	<0.05	<0.05	<0.01
60	<0.0001	<0.0001	<0.0001	<0.001

## Discussion

The results of this study show that rTMS delivered at high frequency over the left DLPF cortex is able to inhibit responses to capsaicin-induced pain in healthy subjects. Left DLPFC stimulation showed to exert antinociceptive effects on both right and left hands. The effect seemed to be specific because high-frequency rTMS of the contralateral homologous cortical region (right DLPFC) was completely ineffective on pain measures. To our knowledge, this is the first evidence that hf rTMS given 10 or 20 min after capsaicin application on left DLPFC induces a significant bilateral anti-nociceptive effect on capsaicin pain model in healthy subjects.

The majority of the reports on modulation of pain neural network have principally targeted motor cortex [7–13]. Recently, however, other cortical areas and in particular DLPFC stimulation showed significant effect on pain control [19–28]. Indeed, DLPFC appears to be a potential candidate region to modulate the experience of pain given that it is a critical structure for working memory and attention functions [32, 33]. The relevance of DLPFC in pain modulation and control has been particularly raised among others by Lorenz et al. [19, 20]. They claimed that this cortical area may have a ‘‘top–down’’ mode of inhibition of neuronal coupling along the ascending midbrain–thalamic–cingulate pathway through descending fibers from the prefrontal cortex. Recently, this hypothesis received experimental support by MRI studies of neural connection with the technique of diffuse tensor imaging that revealed anatomical connections between prefrontal cortices and brainstem structures known for their role in pain modulation like periaqueductal gray and nucleus cuneiformis [34].

In agreement with our results, the role of left prefrontal cortex activation in pain control has been recently reported by Borckardt et al. showed that antinociceptive ability of hf rTMS on this area in healthy subjects [23] and in patients with post-operative [26] and neuropathic pain [27]. The antinociceptive effects of left DLPFC were also showed by tDCS technique through stimulation of this area with anodal activating currents in healthy subjects [28].

These papers, however, explored pain reduction only on the body area contralateral to the brain area stimulated (somatotopic), and are only partly comparable with our results as they did not evaluate potential bilateral effects of the brain stimulation performed. Only two studies, those by Graff-Guerrero et al. [24] and by Nahmias et al. [25], have till now explored this topic performing bilateral evaluation of antinociceptive effects of cortical (M1 and DLPFC area) stimulation. Graff-Guerrero et al. [24] investigated the effect of right and left DLPFC rTMS on pain induced by cold pressor test in healthy subjects, and found a bilateral antinociceptive effect of low-frequency right DLPFC stimulation on both hands. This could seem in contrast with our results, as we used hf rTMS that is known to have activatory effects and found antinociceptive effects by left DLPFC. However, it could be argued, according to the theory of interhemispheric rivalry, that following depression of the right side, an indirect activation of the opposite left side DLPFC occurred (through removal of transcallosal interhemispheric inhibition) in a way similar to what we tried to induce by directly performing hf rTMS over left DLPFC.

This also appears in agreement with the results reported by Tamura et al. [12] in a study that explored antinoceptive effect of rTMS on capsaicin-induced pain. These authors were able to induce pain reduction by 1 Hz rTMS over right M1 and documented through SPECT imaging a decreased activation of the right DLPFC together with an increased activation of contralateral motor and prefrontal cortices. Moreover, as we showed in a recent paper, left DLPFC activation was also able to exert control over motor cortical excitability restoring intracortical inhibition that had been reduced by capsaicin application [35].

With regards to the relationship between motor and prefrontal cortices in pain modulation, Graff-Guerrero et al. [24] compared the effect of DLPFC and M1 stimulation and found that differently from DLPFC, motor cortex is able to exert only contralateral control on pain. This would point towards a more general role for DLPFC in pain control in agreement with the top-down model proposed by Lorenz et al. [19, 20]. This view, however, has been recently challenged by the results of Nahmias et al. [25] that found bilateral analgesic effects not only by DLPFC, but also by M1 stimulation, performing high-frequency instead of slow rTMS on the right DLPFC in healthy subjects. These differences do not appear easy to explain: in our opinion, a critical role may have been played by the different methodological approaches, as it is known that varied effects of DLPFC rTMS on acute pain may be influenced by the type of experimentally induced pain. Indeed, pain elicited by capsaicin and mediated by activation of C-fiber pathways has been shown to be reduced by slow rTMS of right motor cortex [12], whereas the same rTMS procedure has been shown to increase the acute laser-implemented pain primarily involving A delta fibers [13]. Under this respect, the results by Nahmias et al. [25] could be explained by the different pain induction technique (thermal stimulation) likely involving more A delta than C-fibers activation. Indeed, if we speculate that right DLPFC is involved in control of pain arising by A delta fibers activation, then activation by fast rTMS of this area could reduce pain [25], while inhibition through slow stimulation would increase it [13]. Unfortunately, Nahmias et al. [25] performed only stimulation of right DLPFC, so we do not know the potential antinociceptive effects of left side DLPFC rTMS in their experimental pain paradigms. However, inference about this issue could be given by Borckhardt et al. [23] that used similar nociceptive induction as Nahmias, but performed activation of the opposite (left) DLPFC obtaining similar antinociceptive effect. Therefore, it should be argued that at least for this pain type, both left and right DLPFC could exert effective analgesic effects. With regards to this point could be interesting the observation by a recent rTMS study (even if not strictly related to pain control) that both left and right DLPFC are needed to induce an effective placebo analgesia phenomenon [36].

Other important methodological aspects that could affect the response to rTMS could concern the intensity and duration of pain stimulation and its ability to activate the descending nociceptive inhibitory control system (DNIC). Nahmias et al. [25] were not able to induce DNIC activity, as they recorded no changes in RIII (a measure of DNIC activation); whereas cold pressor test [24] that give a more intense pain, and capsaicin that induce continuous painful stimulation, are more likely to activate DNIC [20, 37]. In this regard, DNIC has also been shown to be modulable by cortical structures such as DLPFC, which is involved in pain control and in phenomena of pain expectation and placebo [36, 38, 39].

Taken together, these data would suggest that antinociceptive DLPFC activation would involve more right or left side depending on the different qualitative and quantitative aspects of pain. However, several controversial aspects remain and more evidence is needed concerning the effects of right versus left DLPFC and of slow versus fast rTMS in different experimental pain conditions, to evaluate the existence and role of DLPFC interhemispheric differentiation and/or interaction and the relationship with motor cortex in pain processing and control.

As to the timing of magnetic stimulation, in our study, we obtained significant antinociceptive effect by giving rTMS at both 10 and 20 min after capsaicin application. This timing was chosen because subjects reported pain at 10 min after capsaicin application and the increased VAS values became significant at 20 min. At both rTMS delivering times, the antinociceptive effect began just after magnetic stimulation and remained stable across the observation period (60 min). In our opinion, this could have different explanations: it could be due to the lasting effects of rTMS or to the fact that 10–20 min after capsaicin application could represent a critical time window for activation of antinociceptive mechanisms. The fact that significant antinociceptive effect begins after 40 min from capsaicin application for both 10′- and 20′-rTMS is not easy to explain. It could be that 40 min represents a critical time point (as concerns magnitude of VAS score difference) to observe a statistical significance of 10′- and 20′-rTMS effects with respect to capsaicin alone condition.

In conclusion, our findings support the role of DLPFC on nociceptive modulation and control and point towards the opportunity to further investigate the activation of left and right DLPFC in pain processing, with the final aim to optimize strategies for potential therapeutical application in pain conditions.
